# Characterization of Dendritic Cell-Derived Extracellular Vesicles During Dengue Virus Infection

**DOI:** 10.3389/fmicb.2018.01792

**Published:** 2018-08-06

**Authors:** Sharon de T. Martins, Diogo Kuczera, Jan Lötvall, Juliano Bordignon, Lysangela R. Alves

**Affiliations:** ^1^Laboratory of Gene Expression Regulation, Carlos Chagas Institute, FIOCRUZ, Curitiba, Brazil; ^2^Laboratory of Molecular Virology, Carlos Chagas Institute, FIOCRUZ, Curitiba, Brazil; ^3^Krefting Research Centre, University of Gothenburg, Gothenburg, Sweden

**Keywords:** extracellular vesicles, dengue, infection, neglected diseases, RNA sequencing

## Abstract

The dengue virus (DENV), transmitted by *Aedes* spp. mosquitoes, is one of the most important arboviral infections in the world. Dengue begins as a febrile condition, and in certain patients, it can evolve severe clinical outcomes, such as dengue hemorrhagic fever (DHF) and dengue shock syndrome (DSS). The reasons why certain patients develop DHF or DSS have not been thoroughly elucidated to date, and both patient and viral factors have been implicated. Previous work has shown that a severe immune dysfunction involving dendritic cells and T cells plays a key role in increasing the disease severity, especially in secondary heterologous infections. Extracellular vesicles (EVs) are membranous particles that are secreted by several cell types involved in homeostatic and pathological processes. Secretion of EVs by infected cells can enhance immune responses or favor viral evasion. In this study, we compare the molecular content of EVs that are secreted by human primary dendritic cells under different conditions: uninfected or infected with DENV3 strains isolated from patients with different infection phenotypes (a severe case involving DSS and a mild case). Human monocyte-derived dendritic cells (mdDCs) were infected with the dengue virus strains DENV3 5532 (severe) or DENV3 290 (mild), and the EVs were isolated. The presence of cup-shaped EVs was confirmed by electron microscopy and immunostaining with CD9, CD81, and CD83. The RNA content from the mdDC-infected cells contained several mRNAs and miRNAs related to immune responses compared to the EVs from mock-infected mdDCs. A number of these RNAs were detected exclusively during infection with DENV3 290 or DENV3 5532. This result suggests that the differential immune modulation of mdDCs by dengue strains can be achieved through the EV pathway. Additionally, we observed an association of EVs with DENV-infectious particles that seem to be protected from antibodies targeting the DENV envelope protein. We also showed that EVs derived from cells treated with IFN alpha have a protective effect against DENV infection in other cells. These results suggested that during DENV infection, the EV pathway could be exploited to favor viral viability, although immune mechanisms to counteract viral infection can also involve DC-derived EVs.

## Introduction

Flaviviral infections, such as dengue and zika, are transmitted by *Aedes* spp. mosquitoes and are the most important arthropod-borne diseases worldwide (Paixão et al., 2018). Dengue affects 50–100 million people annually, and 40% of the world population is at risk (World Health Organization [WHO], 2016b). This number is greater when including the asymptomatic and mild cases, which are important for maintaining viral transmission (Bhatt et al., 2013). Recently, an increase in transmission and severity has been observed in endemic areas, as well as its reintroduction in locations where the disease was previously eradicated and its extension to new regions, such as southern Europe and North America (review by Wilson and Chen, 2015).

Dengue starts as a febrile disease with retro-orbital pain, headache, nausea and vomiting and can evolve to dengue hemorrhagic fever (DHF) with severe bleeding and organ impairment (World Health Organization [WHO], 2016a), culminating in dengue shock syndrome (DSS), when plasma leakage leads to patient death (Srikiatkhachorn, 2009). The mechanisms driving the progression of DF to DHF are not fully understood to date. DHF is a severe immune dysfunction that includes mechanisms of lymphocyte activation, such as antibody dependent enhancement (ADE) (Halstead and O’Rourke, 1977) and original antigenic sin (Mongkolsapaya et al., 2003). A massive secretion of cytokines by immune cells (cytokine storm) also occurs, and these cytokines can contribute to endothelial cell activation and apoptosis (Limonta et al., 2007; Dalrymple and Mackow, 2012) and induce plasma leakage, the main clinical outcome of DHF. DHF is more common in secondary heterologous infections (Halstead and O’Rourke, 1977) when a response that was triggered against the first infection is not fully protective against the second serotype. This type of cross-reaction can also occur between DENV and different flaviviruses, such as Zika (Barba-Spaeth et al., 2016; Dejnirattisai et al., 2016; Bardina et al., 2017) and yellow fever virus (Moran et al., 2008).

The development of DHF and DSS depends on many factors (Sierra et al., 2006; Long et al., 2009) and differences between viral strains (Leitmeyer et al., 1999). In a previous study, the functional differences between the two DENV strains used in this work, DENV3 290 and DENV3 5532, were shown by our group. DENV3 290 was isolated in 2002 from a patient with primary mild dengue fever in Rio de Janeiro, Brazil, (22°57 S and 43°12 W), while DENV3 5532 was isolated in 2007 in Paraguay (Asunción metropolitan area; 25°35 S and 57°65 W) from a patient with acute primary dengue fever with visceral manifestations that culminated in death (Silveira et al., 2011). Despite small differences in the genome of these strains, DENV3 5532 infection triggers the secretion of proinflammatory cytokines and the death of mdDCs at higher levels compared to infection with DENV3-290 (Silveira et al., 2011).

Dendritic cells (DCs) are antigen-presenting cells (APCs) and are potent phagocytes that have a crucial role in linking the innate and adaptive immunity (Steinman et al., 1975). After capturing antigens, DCs can process and present them to T lymphocytes through MHC, increasing the expression of surface molecules related to antigen presentation (Watarai et al., 2005). Activated DCs can also secrete several cytokines that help to counteract the infection, regulate the proliferation, and trigger the maturation and activity of B lymphocytes (Dubois et al., 1997; Jego et al., 2003) and modulate the activity of NK cells. In addition to cytokine secretion, the transfer of DC-derived extracellular vesicles has been shown to have potent immunological functions (Shenoda and Ajit, 2016). They are able to present antigens and induce the secretion of proinflammatory cytokines (Nolte-’t Hoen and Wauben, 2012), can be transferred to lymphocytes helping to regulate their activity (He et al., 2007; Buschow et al., 2009) and also induce *trans*-infection of lymphocytes, as shown for HIV (Izquierdo-Useros et al., 2010).

Extracellular vesicles are small membrane structures secreted by a wide range of cell types and are detected in almost all biofluids (Raposo and Stoorvogel, 2013). Recently, several groups have demonstrated their role in intercellular communication during homeostatic and pathogenic conditions (Simons and Raposo, 2009). EVs can transfer proteins and several types of RNA between cells, and their composition was shown to be altered under different pathogenic conditions, including viral infection (Schorey et al., 2015). In addition to transferring cytokines, microRNAs and mRNAs to recipient cells, dendritic cell-derived EVs can independently carry out antigen presentation (Utsugi-Kobukai et al., 2003).

Based on the differences between the two isolated strains of dengue virus serotype 3 (DENV3-290 and DENV3-5532), the serious immune dysfunctions observed in DHF, and the importance of DC-derived EVs for immune regulation in general, we investigated whether mdDCs infected with DENV produced EVs and if the content of the EVs secreted by 290-mdDCs and 5532-mdDCs differed. We also investigate whether immune cell-derived EVs have a protective or enhancing effect on the dengue virus infection.

## Materials and Methods

### Generation of Monocyte-Derived Dendritic Cells

Peripheral blood of healthy donors was collected by venous puncture after obtaining informed consent from the donors and approval from the FIOCRUZ Research Ethics Committee (number 514/09). The peripheral blood mononuclear cells (PBMCs) were purified in lymphocyte separation medium (Lonza, Walkersville, MD, United States) following a previously described protocol (Böyum, 1968). The CD14^+^ monocytes were separated from PBMCs by magnetic immunoaffinity capture using the MACS system (Miltenyi Biotec, Auburn, CA, United States) and were incubated for 6–7 days in RPMI 1640 medium supplemented with 50 ng/ml interleukin 4 (IL-4), 25 ng/ml granulocyte macrophage colony stimulation factor (GM-CSF) (PeproTech, Rocky Hill, NJ, United States), 10% fetal bovine serum (FBS) (Gibco, Grand Island, NY, United States), 100 IU/ml penicillin, 100 μg/ml streptomycin and 2 mM L-glutamine (Gibco).

### Immunophenotyping of mdDCs

After monocyte (CD14^+^/CD11c^-^) differentiation to dendritic cells (CD14^-^/CD11c^+^), the cells were immunophenotyped with anti-CD14 (FITC), anti-CD11c (PE) and anti-HLA-DR (APC) to confirm cell purity. After the infection of mdDCs, the cells were immunophenotyped again with anti-CD40 (APC), anti-CD80 (PE-Cy5), anti-CD86 (PE) and anti-HLA-DR (PE-Cy7) (BD Biosciences, Franklin Lakes, NJ, United States) to verify their activation status. The presence of intracellular envelope (E) protein after the infection was determine using a cytoperm/cytofix kit (BD) and a monoclonal antibody against the dengue virus envelope (E) protein (4G2; ATCC^®^ HB-112^TM^). All flow cytometry data were acquired with a FACS Canto II cytometer (BD Biosciences) at the flow cytometry facility at Carlos Chagas Institute-Fiocruz/PR (RPT08L PDTIS) and were analyzed using FlowJo and Prism Graphpad software.

### Viral Strains and Stocks

The viral strains used in this work were isolated from clinical cases, and the genomic sequences are available at GenBank under the accession numbers HQ235027 (DENV3-5532) and EF629369 (DENV3-290). The viral stocks were generated by infection of C6/36 cells at MOI 0.05 with collection of the culture supernatants after 6 days, always keeping the working stocks at a low passage number (3–4). Titration of the viral samples was performed by a focus formation assay, as previously described (Desprès et al., 1993), using the antibody 4G2.

### Infection of mdDCs

The mdDCs were incubated with dengue virus at an MOI of 10 for 2 h at 37°C. This MOI was shown to have better infection rates and immune stimulation than MOI 1 in mdDCs, in a previous work from Silveira et al. (2011) for DENV3 290 and DENV3 5532 strains, and for this reason it was chosen for this study. After the incubation, the inoculum was removed by centrifugation and the cells were incubated for 72 h with RPMI 1640 supplemented with 10% exosome-depleted FBS, 100 IU/ml penicillin, 100 μg/ml streptomycin and 2 mM L-glutamine (Gibco). The number of DV-infected cells was then evaluated by flow cytometry (Silveira et al., 2011) and the supernatant was collected for EV isolation.

### Immunostaining for Intracellular Detection of DENV

For the intracellular staining, cells were blocked for 30 min in PBS containing 5% FBS and 1% human AB serum (Lonza). After blocking, the cells were incubated for 20 min in Cytoperm/Cytofix (BD Biosciences, Franklin Lakes, NJ, United States) protected from light. The cells were then washed with Permwash (BD Biosciences), incubated with FITC-conjugated 4G2 antibody for 30 min at 37°C cells and washed again with PBS. Finally, the cells were recovered in PBS and analyzed by flow cytometry.

### Quantification of Viral Propagation by Titration

DENV3-290 and DENV3-5532 titers were determined by focus formation assay, modified from the protocol described in Desprès et al. (1993). C6/36 cells (*Aedes albopictus*) were kept in Leibovitz’s-15 media (L-15) supplemented with 5% FBS, 0.26% tryptose and 25 μg/ml gentamicin and plated 1 day prior to the assay. Serial dilutions of the DENV strains were used to infect C6/36 for 60 min at 28°C in L-15 media without FBS. After inoculum removal, the cells were incubated with supplemented L-15 media containing 1.6% CMC (Sigma-Aldrich) in a static incubator at 28°C for 7 days. The media was then removed, the cells were washed three times with 1× PBS, fixed with 3% paraformaldehyde (Sigma-Aldrich) for 20 min, and washed again three times with 1× PBS. Permeabilization was performed with Triton X-100 0.5% (Sigma-Aldrich, St. Louis, MO, United States) for 4 min, followed by washing with 1× PBS (3×) and incubation with 4G2 (1:100) for 45 min at 37°C. After washing, the cells were incubated with AP-conjugated anti-mouse (Promega, Madison, WI, United States) at 1:7,500 for 30 min at 37°C. After additional washing (3× with 1× PBS) the reaction was developed using AP buffer containing 6.6% NBT and 3.3% BCIP. The average for each dilution was calculated between the duplicates, and the amount of focus formation units per ml (FFU_C6/36_/ml) was calculated.

### Production of EV-Depleted FBS

All the FBS used for EV harvesting was ultracentrifuged at 28,000 RPM for 18 h. The supernatant was carefully collected so as not to disturb the vesicle pellet, 0.22 μm filtered, and used to supplement the cell culture media.

### Isolation of EVs From Culture Supernatants

This protocol was adapted from the isolation protocol described by Lässer et al. (2012). The culture supernatants of mdDCs infected with DENV or mock controls were centrifuged at 2,500 × *g* for 5 min and 0.22 μm filtered to eliminate large vesicles and apoptotic bodies. The material was ultracentrifuged for 70 min at approximately 100,000 × *g* in a Hitachi P28S swinging bucket rotor, and the samples were then resuspended in sterile-filtered PBS and centrifuged again. The EVs were then carefully recovered in 100 μl of PBS, aliquoted and stored at -80°C. All the isolations were performed under sterile conditions.

### Detection of Tetraspanins in EV Samples by Electron Microscopy

The samples were processed for electron microscopy with a protocol modified from Lässer et al. (2012). A collodion-coated nickel grid was incubated for 60 min with the vesicles diluted in PBS and then washed three times in PBS. After fixing with 2% paraformaldehyde for 20 min, the samples were washed again, blocked in 1% BSA for 30 min, and incubated with the primary antibody (anti-CD9, anti-CD63, anti-CD81, or DV anti-E, 4G2). After washing, the grids were incubated for 1 h with anti-mouse conjugated with 10 nm gold particles and washed. After post-fixation with 2.5% glutaraldehyde, the samples were washed with deionized water and stained with 0.4% uranyl acetate for 10 min. The grids were examined with a JEOL JEM-2100 TEM microscope at the electron microscopy facility of the Federal University of Santa Catarina (Florianópolis, SC, Brazil).

### Detection of Tetraspanins in EV Samples by Flow Cytometry

To allow the flow cytometry analysis, EVs were conjugated to 4-μm aldehyde-sulfate latex beads and stained for the EV-enriched molecules CD9, CD63, and CD81 following a previously described protocol (Lässer et al., 2012). First, each sample of 3 × 10^7^ beads was conjugated with 12.5 μg of mouse anti-human HLA-DR antibody (capture antibody) and incubated for 16 h at RT, washed 3× in PBS and stored in sterile glycine 0.1% buffer (pH 7.2) prior to use. Each EV sample (representing a pool of EVs from three different donors) was then incubated with 10^5^ beads for 16 h at 4°C (under agitation), blocked with 200 mM glycine for 30 min and washed twice. The Fc receptors that were possibly present on the EVs were blocked with mouse IgG for 30 min at 4°C. After washing, the samples were incubated with 10 μl of anti-CD63, anti-CD81 or anti-CD9 conjugated with PE, diluted in 100 μl of PBS. As controls for this experiment, we stained beads that were incubated with ultracentrifuged supernatants instead of resuspended pellets (control 1: SNT), incubated beads with “vesicles” isolated from unconditioned media supplemented with exo-depleted FBS (control 2: EV media), and we also had a control sample that underwent all the incubation steps except incubation with vesicles (control 3: EV). After 40 min of incubation, the samples were washed again and resuspended in 300 μl of filtered PBS prior to analysis in a FACSCanto II flow cytometer (BD Biosciences). The flow cytometry results were analyzed using FACS DIVA v 6.0 software (BD Biosciences) and FlowJo v10 (TreeStar, Inc.). Statistical analysis was performed in Graphpad Prism (GraphPad version 5.0c, San Diego, CA, United States).

### EV Collection for RNA Sequencing

Monocyte-derived dendritic cells from six different donors were differentiated as described and infected with mock (control), DENV3-5532, or DENV3-290 (MOI 10) for each donor. After 72 h, the cell supernatants were collected, pooled and processed for EV isolation.

### RNA Isolation, Pre-sequencing Analysis and Quantification

After the isolation, the small RNAs and RNAs bigger than 200 nt were extracted from the EV samples from six different human mdDC donors using a mirVANA RNA isolation kit, according to the manufacturer’s recommendations. Size, quality and quantification of the isolated RNAs were assessed on an Agilent 2100 Bioanalyzer using the RNA 6000 pico kit (Agilent Technologies, Santa Clara, CA, United States).

### RNA Sequencing and Data Analysis

Extracellular vesicle-derived RNAs were processed for SOLiD sequencing according to the manufacturer instructions, and the experimental design and analysis was made according to Auer and Doerge, 2010. RNA sequences were mapped against the human reference genome v18 and the viral genomes using CLC Workbench^TM^ 5.1 in the next-generation sequencing mode. The following settings were used: 1 point for each match, each mismatch would subtract 2 points, and each base insertion or deletion would subtract 3 points. With this setting applied, 80% of the reads (0.8) had to map with a similarity greater than or equal to 90% (0.9) of the annotated genome. Sequences that did not map in the viral genomes were mapped against the human genome using the same parameters. Statistical comparisons were made using the KAL-z test (Kal et al., 1999) generating an FDR from corrected *p*-values. Genes with FDRs smaller than 5% were selected for further analysis. The RNA-seq data are deposited in the NCBI Sequence Read Archive database (SRA) under the accession number SUB3741218.

### Analysis of Targets of miRNAs Found in EVs

Analysis of miRNA targets was performed using *Diana mirPATH 3.0* (Vlachos et al., 2012), with microT CDS database, using the “pathways union” option. This tool calculates target mRNAs for each miRNA in the subset, and after that performs an enrichment analysis on this mRNA population using KEGG integration, providing a *p*-value for each alignment with putative target pathways for a given miRNA subset.

### Pathway Enrichment Analysis for mRNAs Identified in EVs

Genes identified with zero expression values in mock EVs and expressed at lest in one infective condition (DENV3-290 and/or DENV3-5532), and with FDR < 0,05 were analyzed with the software Ingenuity Pathway Analysis, to identify pathways enriched in each subset. Pathways with positive fold change (*z*-score) are more expressed in DENV3-5532 EVs, while pathways with negative fold changes were more expressed in DENV3-290 EVs. This software also shows graphs for pathways, telling if a given gene would activate or deactivate a certain pathway.

### Validation of the RNAseq Experiment With RT-PCR of EV RNAs

Extracellular vesicles secreted by infected or uninfected DCs were collected from three human donors, different from the original sequencing, and their RNA was extracted as described in Section “RNA Isolation, Pre-sequencing Analysis and Quantification.” After a reverse transcription reaction (same as section “RT-PCR of Viral RNA After Depletion With Magnetic Beads”) the genes were amplified by PCR with 5 pmol of the following primers for each gene: PSMB3 (F TGGTGGCCAACCTCTTGTAT and R AAGGGCTTAAAGGTCTTCGG), CRIP1 (F AAGTGTCCCAAGTGCAACAA and R GTCAGCGTCTTCCCACATTT), NDUFB7 (F CCTGCAGATGCCAACCTT and R GCGTCCATCATCTCCTGCT), TXNL4A (F TCCTACGTGCATGAAGATGG and R TGTTGAAGTCAGGCACTTCTG), GRHPR (F ACCACTGCCTACAAACCACC and R CAACAAGGACATGGTGTTGC), RPS13 (F ACGACGTGAAGGAGCAGATT and R ACTTGTGCAACACCATGTGAA), GSTT1 (F TGTGGATGAGTACCTGGCAT and R GTGTCTGGGGAGATACTGGC), COX7C (F GTATGTTGGGCCAGAGCATC and R TTGTTTTCCACTGAAAATGGC). Reactions were incubated using the following cycle: 50°C 2′; 95°C 10′; repeating the stages of 95°C 15″, 60°C 1′ and 72°C 1′ for 40 cycles. The samples were analyzed in 1% agarose gel stained with ethidium bromide. For primer construction, the mRNAs were confronted with the databank of *q primer depot* (Cui et al., 2006), and genes found exclusively in each condition (mock, 290 or 5532) were selected.

### Depletion of Viral Particles From Viral Stocks and EV Samples

For the depletion experiments, 10 μg of purified 4G2 antibody was coupled to 5 × 10^8^ anti-mouse IgG magnetic beads (10 μl, New England Biolabs) following the manufacturer’s instructions. The beads were then incubated with samples of viral stocks or mdDC-derived EVs for 60 min at 4°C, and the supernatants were magnetically separated from the beads. Ten microliters of these supernatants (submitted to bead incubation) were collected for titration, and the remaining supernatant was depleted again in another round of incubation with beads, completing three rounds of depletion per sample.

### RT-PCR of Viral RNA After Depletion With Magnetic Beads

The extracted EV RNA was used for reverse transcription (5,6 μl of each sample) with ImProm-II TM Reverse Transcription System (Promega) following manufacturer’s instructions, using 2,5 μl of Random primer (invitrogen) at 100 pmol/μl. Then the cDNA was submitted to a nested PCR protocol, specific for dengue virus, in a 2 step PCR with specific primers for DENV serotype III, a modified protocol based on Lanciotti et al. (1992). Gel bands were quantified in ImageJ and the values for each band were converted into percentages of the input expression, to plot graphs using Graphpad Prism.

### Isolation of EVs From Cells Treated With Interferon Alpha (IFN-α)

Peripheral blood mononuclear cells were collected from healthy donors as described above and seeded in RMPI 1640 supplemented with 10% EV-depleted FBS, 100 IU/ml penicillin, 100 mg/ml streptomycin and 2 mM L-glutamine (Gibco). After 24 h of resting, the cells were treated with 100 IU/ml of IFN-α 2A (Blau Farmacêutica, Cotia, SP, Brazil). After variable incubation times (1:30 h, 3 h or 24 h), the cell supernatants were collected for EV isolation, as described above. Next, new cells from the same donors were infected with DENV at an MOI of 10, as described above. EVs from 10^7^ IFN-treated PBMCs were then incubated with 10^6^ infected cells from the same donor for 72 h. The cells were then analyzed for the intracellular presence of the viral E protein. The ultracentrifugation supernatants of the IFN-treated samples were used to treat the infected cells as negative controls. This would exclude the possible biological effect of free IFN present on the EV preparations, originated from possible vestiges of ultracentrifugation supernatants when the EV pellets were resuspended. Percentages of infection were normalized to 100% for each donor, and compared with the percentages observed after the treatments using one-way ANOVA.

## Results and Discussion

### DENV-3 5532 Cause Productive Infection and Activation in Human Monocyte-Derived Dendritic Cells

**FIGURE 1:**
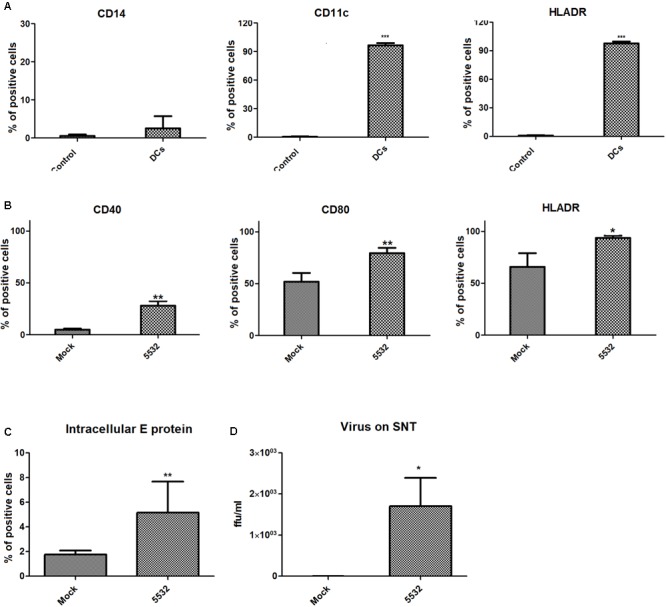
Monocyte-derived dendritic cells express surface activation markers and intracellular E protein after infection with DENV. **(A)** Expression of mdDC markers after monocyte differentiation to dendritic cells. The expression of CD14, CD11c, and HLADR was analyzed after treating CD14+ cells with IL4 and GMCSF in cells from three different human donors. **(B)** Expression of activation markers on mdDCs after DV-infection. The expression of CD40, CD80, and HLA-DR was analyzed on DENV and mock-infected cells after 72 h. Statistical differences were evaluated using *T*-tests, using cells from three different human donors (^∗^*P* < 0.05, ^∗∗^*P* < 0.01, ^∗∗∗^*P* < 0.0001). **(C)** Intracellular detection of viral E protein in mdDCs after 72 h of infection. Expression levels of the viral E protein in mdDCs infected with DENV3-5532 (MOI 10, 72 h) were compared with the expression in Mock-infected cells using *T*-test (^∗∗^*P* = 0,0082), in cells from six different human donors. **(D)** Detection of viable viral particles on the culture supernatants of infected mdDCs. Titration of viral particles in the cell supernatants was performed by focus forming assay, through the detection of viral E protein. Results from infected and mock cells from three different donors were analyzed using *T*-test (^∗^*p* = 0.0134), and are represented in Log10 FFUc636/ml.

Immune cells are important biological targets of infection by dengue virus, especially dendritic cells (Ho et al., 2001). Unfortunately, immortalized lineages of dendritic cells are not good models for evaluating the mechanisms modulated during infection. The differentiation of primary human CD14^+^ monocytes into mdDCs was assessed by flow cytometry based on the expression of CD11c^+^ and HLA-DR^+^ on the cell surface and the reduction of the expression of CD14^+^ molecules, in agreement with the protocol established by Sallusto and Lanzavecchia (1994) (**Figure 1A** and Supplementary Figure S1).

DENV3-5532 is a serotype 3 strain that belongs to genotype III (Sri Lanka) (Pan-American Health Organization [PAHO], 1995) and was isolated in 2007 during an outbreak of severe dengue in Paraguay. The patient presented an acute dengue infection with severe clinical complications culminating in DSS 5 days after the onset of symptoms (Silveira et al., 2011). The other strain of DENV3 used in this study, DENV3-290, which also belongs to genotype III (Sri Lanka) (Pan-American Health Organization [PAHO], 1995), was isolated from a dengue fever case in Brazil, which resolved without further complications. The differences between the two strains consist of 14 amino acid substitutions targeting the prM, C, E, NS1, NS2B, and NS5 proteins and the 3-UTR region of the viral RNA (Silveira et al., 2011). In addition, mdDCs were more susceptible to infection with DENV3-5532 when compared to DENV3-290. It was also shown that the DENV3-5532 strain (fatal case) was able to induce a more pronounced pro-inflammatory response than DENV3-290 (dengue fever). A multiplicity of infection (MOI) of 10 induced these effects more effectively than an MOI of 5 or 1 (Silveira et al., 2011). Based on previous studies, mdDCs were infected with DENV3-5532 and analyzed at 72 hpi. Flow cytometry and the focus-forming assay demonstrated that the DENV3-5532 strain was able to infect and replicate in mdDCs (**Figures 1C,D**). Additionally, DENV3-5532 can trigger mdDC activation, as assessed by the expression of markers such as CD40, CD80 and CD86 on the cell surface (**Figure 1B**). The gates were delimited using isotype controls for each fluorochrome (Supplementary Figure S1).

The activation of mdDCs after infection by dengue virus DENV3-5532 or other strains or serotypes was previously shown (Ho et al., 2001; Silveira et al., 2011). These results are in agreement with previous research, showing the infection and activation of mdDCs by DENV (Ho et al., 2001; Libraty et al., 2001; Palmer et al., 2005) and allowing the next steps of EV collection and isolation from infected and uninfected mdDC culture supernatants.

### Primary mdDCs Infected by DENV Produce Small Extracellular Vesicles (<200 nm) Expressing Tetraspanins

*In vitro* and *in vivo* studies have previously shown that EVs derived from antigen-presenting cells can have potent immunomodulatory functions by amplifying the adaptive immune response (Raposo et al., 1996; Théry et al., 2002; Nolte-’t Hoen and Wauben, 2012). Dendritic cell-derived EVs can be important during viral infections, as is shown for HIV, where dendritic cell-derived EVs can carry HIV particles, leading to *trans*-infection of CD4 T cells and helping in the dissemination of HIV (Muratori et al., 2009; Izquierdo-Useros et al., 2010). For this reason, we decided to investigate the extracellular vesicles that are secreted by DCs during DENV infection.

**FIGURE 2:**
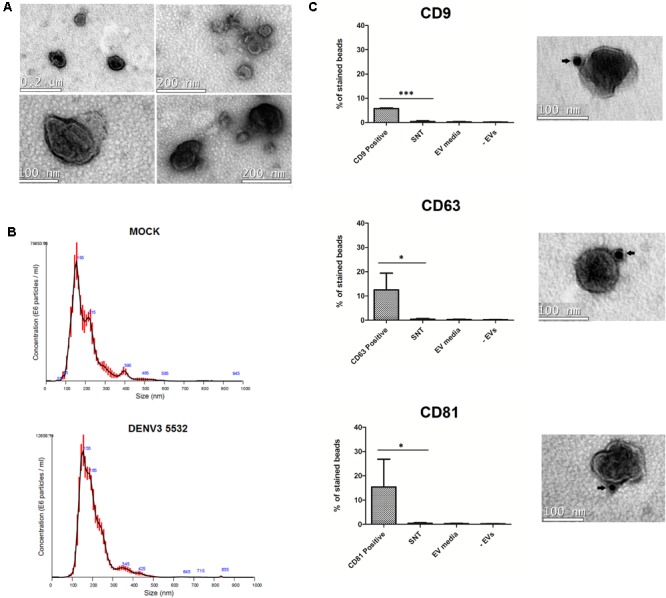
Characterization of mdDC derived extracellular vesicles. **(A)** Vesicles secreted in mdDC culture supernatants have 30–180 nm and present the characteristic cup shaped morphology of EVs. **(B)** Size distribution analysis of EVs from Mock and DENV3-5532 infected cells (MOI 10, 72 h). Most of the vesicles have a distribution range around 100 nm, as described before for EVs. Red lines are an average distribution of five measurements. **(C)** mdDC derived EVs express the tetraspanins CD63, CD81, and CD9 on its surface. The antibody staining with gold particles is indicated by arrows. These markers are also detected on mdDC derived EV populations by flow cytometry, using three pools of samples of three different mdDC donors each (nine samples total), and not detected in three different negative controls. mdDC EVs: EVs from mdDCs infected with DENV3-5532, MOI 10, 72 h; SNT: negative control – ultracentrifugation supernatants after EV pelleting; EV media: negative control – “EVs” isolated from unconditioned media; –EVs: negative control, beads processed the same way as samples, without incubation with EVs. Statistical analysis: one way anova (^∗^*P* < 0.05, ^∗∗^*P* < 0.01, ^∗∗∗^*P* < 0.0001).

The culture supernatants of mdDCs were collected 72 h post-infection, and EVs produced by DENV-infected cells or mock cells were isolated. In agreement with current understanding in the EV field (Lötvall et al., 2014), we used different approaches to confirm the presence of EVs in the samples. After isolation, the extracellular vesicles were morphologically characterized. The cup-shaped morphology (characteristic of EVs observed by transmission electron microscopy after fixation) could be observed for these vesicles (**Figure 2A**). In addition to the cup-shaped morphology, we observed that most of the vesicles depicted in the electron micrographs were smaller than 200 nm in diameter. To further analyze the size distribution of this population of vesicles, we performed nanoparticle tracking analysis of the EV samples (**Figure 2B**). Thus, we confirmed that most of the mdDC-derived vesicles had a size smaller than 200 nm, and the size distribution of the vesicles produced either by cells infected with DENV3 5532 or mock cells had similar patterns (**Figure 2B**).

After the structural analysis, we decided to investigate the expression of the tetraspanins, CD9, CD63, and CD81, which are known to be present in EV populations (Lässer et al., 2012). Two approaches were used to confirm the presence of these markers. The first one was staining EV samples with anti-CD9, anti-CD63 and anti-CD81 antibodies conjugated with gold particles and visualizing the results by electron microscopy. All the three markers were observed on the surface of the EVs produced by the infected mdDCs (**Figure 2C**). The second approach, using latex beads and flow cytometry, allowed the relative quantification of these tetraspanins in mdDC-derived EV samples and also included additional controls to verify the purity of our samples, further validating our EV isolation protocol. Latex beads coated with anti-HLADR antibodies were incubated with EV samples, since HLA-DR is highly expressed by activated mdDCs, and this molecule is known to be present in DC-derived vesicles (Lässer et al., 2012). The presence of three common EV tetraspanins was then evaluated on the EVs bound to the beads. We observed that among the three tested tetraspanins, CD81 was the most abundant in mdDC-derived EVs, followed by CD63 and CD9 (**Figure 2C**). Notably, with the protocol used, it could be concluded that EVs had a double reactivity, recognizing both HLA-DR on the conjugated-beads and each of the tested tetraspanins.

Additional controls were performed to verify whether the observed tetraspanin staining could be due to free proteins present in the ultracentrifugation supernatants (control 1 – SNT), a residual presence of EVs from the exo-depleted FBS (control 2 – EV media) or a false-positive staining due to the direct binding of anti-CD9, anti-CD81 and anti-CD63 to the latex beads (control 3 – EV). The results of control samples (1–3) demonstrated that the protocol used for EV purification was specific and validated the data (**Figure 2C** and Supplementary Figure S2). The characterization experiments were performed prior to viral particle depletion with magnetic beads.

### EVs From DENV-Infected mdDCs Carry miRNA Molecules Associated With Infection, Which Could Be Useful for Diagnosis and Prognosis

The progression of dengue fever to severe clinical outcomes involves a strong and multifactorial immune dysfunction with such mechanisms as antibody dependent enhancement (Halstead and O’Rourke, 1977), original antigenic sin (Mongkolsapaya et al., 2003) and cytokine storm (Pang et al., 1988). DCs have immunomodulatory potential, controlling the activity of B, T, and NK cells, and the differentiation of monocytes into DCs intensifies the response against DENV through lymphocyte activation (Hou et al., 2012). We hypothesized that vesicles from infected DCs loaded with RNAs could help to regulate the activity of other cells, both at a close (i.e., T cells and other DCs) and long distance (i.e., endothelial cells) contributing to the immune dysfunction observed in DHF/DSS. To verify this hypothesis, we isolated EVs derived from mdDCs infected with DENV3 290 (mild dengue case) or DENV-3 5532 (severe infection phenotype) and maintained uninfected controls. After isolation, small RNAs and mRNAs present in EVs were extracted and processed for RNA sequencing (Supplementary Table S1).

**FIGURE 3:**
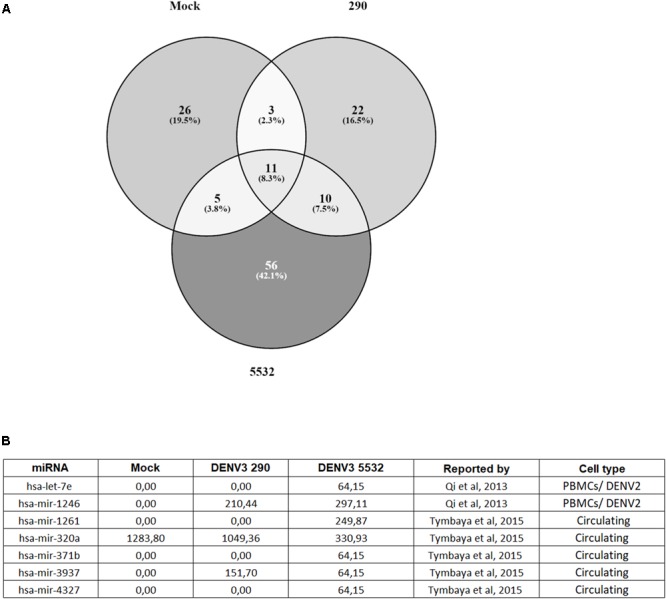
Distribution of miRNAs found in EVs from Mock, DENV3-290 and DENV3-5532. **(A)** miRNAs found in EVs derived from mdDCs infected with DENV3-290, DENV3-5532 or uninfected controls, using cells of six different donors. **(B)** miRNAs found in EV data that are described on the literature as associated with DENV infection. Expression data is represented as normalized expression values.

First, the micro RNAs (miRNAs) found in each condition (DENV3 290, DENV3 5532 and mock) were compared (**Figure 3A**). EVs from mdDCs infected with DENV3-5532 had a higher number of exclusive miRNAs (56) compared with DENV3-290 EVs (22) or mock EVs (26) (**Figure 3A**). Several of the miRNAs that were found exclusively in DENV3-5532 EVs were found to be associated with DENV infection in previous works (let-7e, mir-1261, mir-142, mir-371b, and mir-4327), as were two miRNAs found in both DENV-3 290 EVs and DENV3 5532 EVs. Tambyah et al. (2015) compared the composition of circulating miRNAs in the blood of dengue and influenza patients, and some micro RNAs were found only during DENV infection, while others were also observed during influenza infection. However, only one DENV strain was used in this work, and the cell origin of the circulating miRNAs was not determined (Tambyah et al., 2015). The mir-3937 was noted as a molecule involved with general mechanisms of infection since it was found in influenza and dengue patients. Here, mir-3937 was found in EVs from DENV3-5532 and DENV3-290 cells and not in uninfected controls (**Figure 3B**). Additionally, mir-4327 has been indicated in a previous work as a marker for dengue infection since it could not be detected in influenza patients, and it was also detected in DENV acute infections but not in recovering patients or healthy donors (Tambyah et al., 2015). With our findings on EVs, we believe that mir-4327 can be a promising candidate for future studies about circulating markers of severe dengue, since it was secreted in vesicles by immune cells infected with a hemorrhagic DENV3 strain (DENV3-5532) but not by cells infected with a mild DV strain (DENV-3 290) from the same serotype.

Other highly expressed miRNAs found in patients with acute DENV infection were mir-1261, mir-371b, and mir-3937 (Tambyah et al., 2015). Interestingly, mir-3937 was found to be expressed in DENV3-290 and DENV3-5532 EVs but not in mock EVs. On the other hand, mir-1261 and mir-371b were found in DENV3-5532 EVs but not DENV-3 290 or mock EVs, suggesting that similar to mir-4327, it could be further explored to investigate if it is related to disease severity. Since mir-4327, mir-1261, and mir-371b were previously found in the bloodstream of dengue patients (Tambyah et al., 2015), and they were observed in this study in DENV-infected mdDCs-EVs, this indicates that mdDCs could be the source of these miRNAs in infected patients. The association of these circulating miRNAs with EVs reinforces their potential to further exploration as a prognostic tool, since they would be protected from degradation while circulating in the blood.

Likewise, it was shown that human PBMCs infected by DENV-2 present a 3.2-fold increase in mir-1246 expression compared with mock controls, and let-7e had 8.5-fold increase during infection (Qi et al., 2013). In this study, both miRNAs were associated with EVs from infected cells and not with mock-infected cells (**Figure 3B**). While let-7e was associated only with DENV3-5532, mir-1246 was present in EVs secreted by cells infected with both DENV strains. This result indicates that these miRNAs, that have been reported before to be inside cells infected with DENV, can also be secreted in EVs, reinforcing their potential as circulating biomarkers of disease.

Micro RNAs are small non-coding RNAs involved in posttranscriptional regulation of gene expression. Usually, they target specific mRNAs hindering their translation (Chen and Rajewsky, 2007). Regulation through miRNAs occurs in a combinatorial way: one miRNA can have multiple mRNA targets, while a given mRNA can be regulated by several miRNAs (Krek et al., 2005). Some tools like DIANA mirPATH are able to predict miRNA targets and group them in pathways, shedding light on the possible targets of these miRNAs *in vivo*.

We searched for putative target pathways for EV miRNAs, using DIANA miRPATH 3.0, and some interesting targets are summarized in Supplementary Table S2. The Erb, MAP kinases (MAPK), phosphatidylinositol-3-kinase/Serine-threonine kinase (PIK3/AKT) and phosphatidylinositol pathways seems to be sinergically regulated by miRNAs secreted in EVs from DENV3-5532 infected cells. Erbs are tyrosine-kinase receptors, functionally related to MAPK and PIK3/AK7 pathways, and PIK3/AK7 is related to the mechanistic target of rapamycin (mTOR) pathway (Linggi and Carpenter, 2006). PIK3/AK7 is a cellular survival pathway dependent on extracellular signals. Activation of surface receptors leads to PIK3 phosphorylation, and this activated molecule phosphorylates membrane lipids originating the second messenger phosphatidylinositol, that recruits AKT (Matsuoka and Yashiro, 2014). One of the described effects of activated AKT is related to cellular survival (Osaki et al., 2004). Blocking PIK3/AKT pathway with miRNAs, and also its associated pathways, could collaborate with higher apoptosis rates observed by Silveira et al. (2011). Tambyah and colleagues also showed that PIK3/AKT was the pathway more intensely regulated by DENV-specific miRNAs, followed by the MAPK pathway *in vivo* (Tambyah et al., 2015), corroborating with our results. In addition, miRNAs targeting the transforming growth factor beta (TGF beta) pathway (enriched in DENV3-5532 derived EVs) seems to work in similarly in this model. TGF beta is a known cytokine with anti-inflammatory properties, and is associated with viability of dendritic cell precursors in the presence of tumor necross factor (TNF) (Riedl et al., 1997), and regulation of apoptosis (Schuster and Krieglstein, 2002). Interference with the PIK3/AKT and TGF beta pathways could facilitate dendritic cell apoptosis and the maintenance of a strong pro-inflammatory environment, as observed before for this strain (Silveira et al., 2011).

The WNT pathway, usually associated with cell growth and differentiation, can be manipulated to increase viral survival in hosts, as shown for Epstein barr virus, for the herpesvirus associated with Kaposi sarcoma (Hayward et al., 2006) and for citomegalovirus (Angelova et al., 2012), among others. In dendritic cells, the WNT/Beta catenin pathway can regulate cell differentiation (Swafford and Manicassamy, 2015) and play a pivotal role in regulating the balance between inflammatory and tolerogenic responses (Manicassamy et al., 2010). The probable interference of EV RNAs with the WNT pathway suggested by this experiment could open new possibilities of exploration for the role of WNT during DENV infection.

The “mRNA surveillance pathway” (that involves no sense mediated decay, non-stop mRNA decay and non-go decay), is essential to detect and degrade abnormal RNAs produced in the cell, including viral RNAs. It was shown that several miRNAs present on DENV3-5532 EVs are able to interfere with this pathway, and this could contribute to the greater ability to induce productive infection observed for DENV3-5532 (Silveira et al., 2011). Also, the “Ubiquitin mediated proteolysis” pathway identified in our dataset was previously associated with DENV and influenza infection (Tambyah et al., 2015), and also for human cells infected with Chikungunya (Saxena et al., 2013), suggesting that the inhibition of ubiquitin mediated proteolysis can be a conserved escape from antiviral immune responses, and this survival mechanism could be propagated through EVs.

### EVs Generated During Infection With Different DENV Strains Carry Distinct Subsets of mRNAs

In addition to miRNAs, other RNA types were also found in the EVs, including mRNAs (Kim et al., 2017). We analyzed the mRNAs present in EVs secreted by mdDCs during infection with DENV3 5532, DENV3 290 and by mock cells. Also, using RT-PCR we confirmed the presence of some mRNAs found in EV sequencing (from DENV3-5532, DENV3-290 or mock). Donors that provided blood to generate mdDCs/EVs used for RT-PCR were different from the original donors of cells/EVs used for RNA sequencing in order to validate our findings. The mRNAs confirmed by RT-PCR were RPS13, GSTT1, COX7C (found exclusively in EVs from mdDCs infected with DENV3-5532); GRHPR, TXNL4A, NDUFB7 (found exclusively in EVs from mdDCs infected with DENV3-290) and also PSMB3 and CRIP1 (found only in EVs from Mock mdDCs) (Supplementary Figure S4).

In order to verify patterns in EV-RNA data, we performed an *Ingenuity Pathway Analysis* (IPA) on the full subset of mRNAs present on DENV3-5532 EVs and/or DENV3-290 EVs, but not present in mock EVs (FDR < 5%). The full list of RNAs used for this analysis is presented in Supplementary Table S3. The pathways enriched in DENV3-5532 are presented in the tables with a positive *Z*-score, while pathways enriched in DENV3-290 are presented with a negative *Z*-score. In this analysis, we searched in the “Diseases and functions” and “Canonical Pathways” modes (Supplementary Tables S4, S5), and some interesting information gathered from this analysis is discussed below.

Some pathways related to immune responses, found mostly enriched in DENV3-5532 RNAs, are investigated in more detail in Supplementary Figure S3. For the pathway “Immune response of dendritic cells” we observed that genes enriched in DENV3-290 have an inhibitory activity over this pathway, while one gene enriched in DENV3-5532 EVs have an activation role, corroborating previous findings of intense activation induced by DENV3-5532 (Silveira et al., 2011). For “activation of dendritic cells,” several genes found enriched in DENV3-5532 EVs displayed an activation function over this pathway. For DENV3-5532, we also found RNAs related to migration of Langerhans cell precursors, interestingly, these dermal DCs are usually the first targets of DENV *in vivo* (Wu et al., 2000).

We also found pathways regulating the activity of other immune cells, like chemotaxis and adhesion of mononuclear leukocytes and migration of monocytes. Besides being biological targets of DENV, monocytes have a pivotal role in immune responses during infection (Hotta et al., 1984), secreting inflammatory cytokines. They are circulant and easily recruited to infection sites, and molecules involved in the activation of monocyte chemotaxis were found enriched in DENV3-5532 EVs, in agreement with the increased inflammation already observed *in vitro* for DENV3-5532 and in the clinical manifestation of this disease (Silveira et al., 2011). Monocytes are also important precursors of macrophages and dendritic cells (Auffray et al., 2009) and during infection can also induce the expansion of plasmoblasts (Kwissa et al., 2014), that produce antibodies that could have a neutralizing role or increase disease severity, as explained in the *Antibody Dependent Enhancement* (ADE) theory (Halstead and O’Rourke, 1977). Also, we found in EVs from DENV3-5532 molecules involved in monocyte adhesion, a mechanism that helps the “rolling” movement through the blood vessels (Dejana et al., 1994) and also to induce their differentiation. Several molecules with positive effects on monocyte adhesion were found in DENV3-5532 derived EVs, and only one molecule related to this pathway was found in DENV3-290 EVs (Supplementary Figure S3).

We also found enriched in DENV3-5532 EVs molecules related to the activity of T and B lymphocytes. Activation of T cells is one of the main biological functions of DCs (Guermonprez et al., 2002) and exacerbation on their activity can contribute to an increased pathogenicity, as showed by the “*original antigenic sin*” theory (Mongkolsapaya et al., 2003). The interchange of EVs between DCs and lymphocytes through the immune synapse is already known, and can facilitate the delivery of mRNAs produced by DCs to lymphocytes (van der Grein and Nolte-’t Hoen, 2014). Also, activated DCs can induce differentiation, proliferation maturation and activation of B lymphocytes (Dubois et al., 1997, 1998; Jego et al., 2003), through the production of cytokines like IL-2, IL-6 e IL-12, or other mechanisms (Ubol and Halstead, 2010), and the transfer from DCs could also contribute to this phenomenon. Following the theory of “Antibody Dependent Enhancement” (ADE) (Halstead and O’Rourke, 1977), B lymphocyte activity is important in severe cases of Dengue, through the production of low avidity antibodies that end up facilitating the infection (Halstead and O’Rourke, 1977). Pathways associated with NK cell activation were also found. Activation of NK cells in initial times of infection can help to limit viral replication, reducing pathogenesis (Azeredo et al., 2006). However, the increased expression of MHC I by infected cells can help them to evade the NK cytotoxic response (Beltrán and López-Vergès, 2014). Their role during infection is ambiguous, because they can help to counteract the infection but at the same time inflict tecidual damage. Some ligands of the activation receptor NKG2D present in NK cells (as MICB and MICA) are associated to disease severity in dengue (Beltrán and López-Vergès, 2014). We can also observe Gene Ontology categories related to chemotaxis, activation, proliferation and desensitization of granulocytes, cells that are important during DENV infection like was shown for neutrophils, basophils and mast cells (King et al., 2002; Moreno-Altamirano et al., 2015).

Other interesting pathways showed enriched for DENV-5532 EVs were related to positive regulation of apoptosis; what could contribute to the apoptosis of bystander uninfected cells as previously shown (Silveira et al., 2011), and positive regulation of antiviral and antimicrobial response (consistent with the larger immune activation induced by DENV3-5532). Between the microbial response pathways, we can note iNOS signaling, PRPs, activation of cytoplasmic sensors like RIG-1 and activation of IFN-mediated antiviral response (Supplementary Tables S4, S5). Also, mRNAs related to platelet and endothelial cell activation are enriched DENV3-5532 EVs, besides cytokines associated to plasma leakage and DSS (like IL6). This is interesting due to the hemorrhagic nature of DENV3-5532 infection in patients, which is not observed for DENV3-290 strain. Transcripts of cytokines already correlated to disease severity in dengue patients (like CXCR4, MIF, IL17A e IL8), were found enriched in these EVs from DENV3-5532. Analysis of these pathways can help to comprehend the molecular context of DENV infection, and also demonstrate that several pathways that are important for the course of infection could be potentially regulated by mdDC derived EVs, a mechanism widely described and studied for other infectious conditions, including other viral infections (Hosseini et al., 2013).

The EV-mRNA contents were also compared with the cellular mRNA expression during mdDC infection with DENV3-5532 (Silveira et al., 2011). These correlations confirmed that expressed mRNAs presented in DENV-infected cells were also observed in the EVs. In addition, it would be possible to verify which RNAs could potentially be secreted by mdDCs in EVs with the ability to reach other cells. From this analysis, we found 39 mRNAs (**Table 1**). The majority of the mRNAs are associated with innate immunity, chemokines (CXCL10 and CXCL11), factors related to viral replication, ATP-dependent helicases (DDX58, DDX60, and DDX60L) and interferon-stimulated genes, such as IFI35, IFI44L, IFIT1, IFIT5, IFIT3, IFTM1, which are important effectors of the type I IFN response. From the 39 mRNAs observed in DENV-infected EVs, 31 were found either exclusively or enriched in EVs from DENV3 5532 when compared with EVs of DENV3 290, and 8 were enriched in EVs from DENV3 290 (**Table 1**).

**Table 1:** mRNAs found enriched in mdDCs infected with DENV-3 5532 in a previous work (Silveira et al., 2011) also detected in mdDC derived EVs.

ID	Annotation	290 vs. 5532 fold change	290 vs. 5532 – *p*-value corrected FDR	Normalized expression means 290	Normalized expression means 5532
APOBEC3A	Probable DNA dC- > dU-editing enzyme APOBEC-3A	1.80E+308	0.022	0.000	8.304
BLZF1	Golgin-45	1.80E+308	0.000	0.000	19.258
BTBD11	Ankyrin repeat and BTB/POZ domain-containing protein BTBD11	1.80E+308	0.000	0.000	17.621
CRLF2	Uncharacterized protein	1.80E+308	0.000	0.000	75.314
CSF1	Uncharacterized protein	1.80E+308	0.022	0.000	8.313
CXCL10	C-X-C motif chemokine 10	1.80E+308	0.000	0.000	134.604
CXCL11	C-X-C motif chemokine 11	1.80E+308	0.000	0.000	27.796
DDX58	DEAD (Asp-Glu-Ala-Asp) box polypeptide 58	1.80E+308	0.013	0.000	9.386
DDX60	Probable ATP-dependent RNA helicase DDX60	1.80E+308	0.000	0.000	17.559
DDX60L	Probable ATP-dependent RNA helicase DDX60-like	1.80E+308	0.048	0.000	6.609
EIF2AK2	Interferon-induced, double-stranded RNA-activated protein kinase	1.80E+308	0.006	0.000	11.043
EPSTI1		1.80E+308	0.013	0.000	9.460
GPNMB	Transmembrane glycoprotein NMB	1.80E+308	0.001	0.000	16.139
GYPA	Glycophorin-A	1.80E+308	0.000	0.000	33.703
HERC6	Probable E3 ubiquitin-protein ligase HERC6	1.80E+308	0.030	0.000	7.652
IDO1	Uncharacterized protein	1.80E+308	0.000	0.000	38.529
IFI35	Interferon-induced 35 kDa protein	1.80E+308	0.000	0.000	23.808
IFI44L	Interferon-induced protein 44-like	1.80E+308	0.009	0.000	10.133
IFIT1	Uncharacterized protein	1.80E+308	0.000	0.000	39.978
IFIT3	Interferon-induced protein with tetratricopeptide repeats 3	1.80E+308	0.006	0.000	11.063
IFIT5	Interferon-induced protein with tetratricopeptide repeats 5	1.80E+308	0.001	0.000	14.751
IFITM1	Interferon-induced transmembrane protein 1	1.80E+308	0.000	0.000	41.506
NT5C3	Cytosolic 5′-nucleotidase 3	1.80E+308	0.026	0.000	7.949
PARP9	Uncharacterized protein	1.80E+308	0.032	0.000	7.461
PLSCR1	Phospholipid scramblase 1	1.80E+308	0.002	0.000	13.466
RSAD2	Radical *S*-adenosyl methionine domain-containing protein 2	1.80E+308	0.003	0.000	12.711
SEPP1	Uncharacterized protein	1.80E+308	0.000	0.000	20.263
SLC39A8	Zinc transporter ZIP8	1.80E+308	0.033	0.000	7.413
SPARC	Uncharacterized protein	1.80E+308	0.013	0.000	9.466
XAF1	XIAP-associated factor 1	1.80E+308	0.003	0.000	12.311
SP110	Uncharacterized protein	6.58E+00	0.000	242.916	1598.704
SAMD9	Uncharacterized protein	–3.18E+00	0.010	27.623	8.683
ADAM19	Uncharacterized protein	–3.18E+00	0.008	29.217	9.184
DSG2	Desmoglein-2	–6.36E+00	0.000	33.496	5.264
SLCO5A1	Uncharacterized protein	–1.27E+01	0.000	47.197	3.709
PGAP1	GPI inositol-deacylase	–1.91E+01	0.000	51.117	2.678
SIGLEC1	Sialoadhesin	–5.09E+01	0.000	112.694	2.214
BRIP1	Fanconi anemia group J protein	–1.80E+308	0.000	23.188	0.000
SGPP2	Sphingosine-1-phosphate phosphatase 2	–1.80E+308	0.000	1262.355	0.000

*Transcripts enriched in DENV3-5532 EVs are highlighted in White, while mRNAs enriched in DENV-3 290 EVs are marked in gray.*

Additionally, some of these mRNAs are related to interferon stimulated genes (ISGs), which are a subset of mRNAs transcribed when a molecule of IFN binds to its extracellular receptors (Der et al., 1998). These molecules help to establish adaptive immune responses and can have direct antiviral properties (Schoggins, 2018). Type I IFN is one of the most important antiviral response mechanisms in human cells (reviewed by Teijaro, 2016), and IFN alpha is particularly important during DENV infection (Castillo Ramirez and Urcuqui-Inchima, 2015). It is produced in the beginning of the infection, when viral PAMPs (pathogen activated molecular patterns) are recognized by cellular PRRs (pattern recognition receptors) (Navarro-Sánchez et al., 2005). Type I IFN (alpha and beta) molecules are secreted by infected cells and can bind surface receptors (type I IFN receptors) on infected and bystander cells, triggering the transcription of ISGs (reviewed by Castillo Ramirez and Urcuqui-Inchima, 2015).

These genes usually code for proteins that are important to helping establish antiviral responses, and some of these proteins exhibit direct antiviral properties (Schoggins and Rice, 2011). It can be hypothesized that this could be a pathway that infected cells would use to protect neighboring cells, transferring ISG mRNAs with the potential to be translated in the recipient cells, thereby making them refractory to the infection before the virus is able to spread to them.

Since we identified ISGs transcripts in the RNA-seq data that were in common with mRNAs found inside mdDCs by Silveira et al. (2011) we decided to investigate whether EVs produced by cells treated with IFN alpha could protect untreated cells from DENV infection. To test the hypothesis that EVs can confer IFN-mediated protection to other immune cells, we performed a functional experiment by treating immune cells with IFN for different incubation times so that the cells could produce ISG mRNAs and package them into EVs. We then isolated the EVs produced by these IFN-treated cells. These EVs were then used to treat new cells infected with DENV, and infection rates were compared between the IFN-EV treated and untreated cells.

### EVs From IFN Treated Cells Can Protect Immune Cells From DENV Infection

Type I IFNs (especially IFN-α) are important during DENV infection and can be secreted by human monocytes when these cells are stimulated by DENV (Kurane and Ennis, 1988). IFN secretion can protect cells from viral infection, and it has been shown in several models that the EV pathway could also be used to propagate IFN-mediated antiviral protection. Li et al. (2013) showed that non-permissive liver non-parenchimal cells (LNPCs) treated with IFN-α were able to transfer EVs to permissive hepatocytes, protecting these cells from infection by hepatitis B virus (HBV) (Li et al., 2013). Kouwaki et al. (2016) also showed that extracellular vesicles released by hepatocytes are able to regulate immune responses against HBV, helping to counteract the infection. Another work showed that EVs produced by liver sinusoid endothelial cells (LSECs) stimulated with type I IFNs or type III IFNs were able to control the replication of hepatitis C virus (HCV) in recipient cells (Giugliano et al., 2015). Additionally, Sun and colleagues showed that extracellular vesicles produced by TLR3-activated brain micro vascular endothelial cells (HBMECs) contain several antiviral molecules, including mRNAs and proteins coded by interferon-stimulated genes (ISG15, ISG56, and Mx2). They also showed that these vesicles could be transferred to macrophages, thus promoting antiviral activity (Sun et al., 2016). Based on this literature evidence and because we identified mRNAs coding for ISGs in our RNAseq data, we decided to investigate whether immune cells treated with IFN-α could produce protective EVs in the context of DENV infection.

**FIGURE 4:**
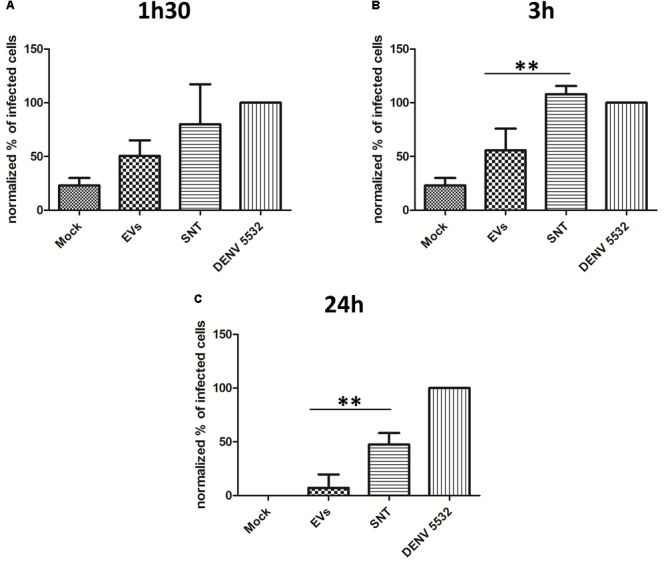
The antiviral effect mediated by Type I IFN can be transferred through EVs. **(A)** Vesicles from cells treated with IFN for 1h30 were collected and used to treat new infected cells. No differences in infection can be seen for this timepoint. **(B)** Vesicles from cells treated with IFN for 3 h were collected and used to treat new infected cells, and EVs can protect treated cells from infection, when compared with cells treated only with the supernatants of ultracentrifugation. **(C)** Vesicles from cells treated with IFN for 24 h were collected and used to treat new infected cells, and even after 24 h post IFN treatement, the secreted EVs can protect treated cells from infection, when compared with cells treated only with the supernatants of ultracentrifugation. Statistical analysis was made using one way anova, with cells of three different human donors (^∗^*P* < 0.05, ^∗∗^*P* < 0.01, ^∗∗∗^*P* < 0.0001). Cells from the same donors were used to collect IFN treated EVs and for the infection experiment. Mock: uninfected controls. SNT: cells infected with DENV3-5532 and treated with ultracentrifugation supernatants; EVs: cells infected with DENV3-5532 and treated with EVs of IFN treated cells; 5532: Cells infected with DENV3-5532 and kept untreated.

Peripheral blood mononuclear cells (PBMCs) were treated with IFN-α for 90 min, 3 h or 24 h. After the different incubation times, culture supernatants were collected and processed for EV isolation. New primary PBMCs from the same human donors were then infected with DENV-3 5532, washed, and the “IFN vesicles” (from 90 min, 3 h or 24 h post IFN incubation) were used to treat the infected cells for 72 h. During the 72 hpi, virus that initially infected the immune cells should complete its replication cycles and spread to other cells. The infection rate was measured by flow cytometry (**Figure 4**). In addition to untreated cells, we used cells treated with ultracentrifugation supernatants (the residual media from EV isolation) as controls, to verify whether the effects observed could be due contaminating free IFN-α in the supernatants. Our results indicated that 90 min of IFN-α treatment was not enough to induce EVs carrying antiviral activity (**Figure 4A**). However, when the cells were treated with EVs derived from the IFN-α treatment for 3 h (**Figure 4B**), it was possible to observe an antiviral effect stronger than from the ultracentrifugation supernatants, suggesting that the effect observed is not due to the free IFN that remained in the supernatant. This effect was still observed with EVs collected 24 h after the initial IFN-α treatment (**Figure 4C**), showing that the secretion of antiviral molecules in EVs can last for hours after the first stimulation with IFN-α. This could be explained by the fact that IFN cascades can self-replicate, leading to the production of more IFN-α in neighboring cells. These results suggest that the EV pathway can be used by immune cells to share defense signals during DENV infection in an attempt to combat viral replication and stop viral infection. Nevertheless, we can still observe productive infection of DENV in immune cells. Dengue virus is known for several mechanisms by which it can evade the antiviral response, allowing it to replicate and successfully complete its life cycle. One of these evasion mechanisms consists of the passive inhibition of the antiviral response by some viruses, avoiding cellular recognition by pattern recognition receptors (PRRs), through the formation of intracellular vesicles and membrane complexes of approximately 90 nm in diameter (Welsch and Zeuzem, 2009) where viral proteins and RNAs can be found (den Boon et al., 2010). Some of these membranes belong to the endoplasmic reticulum and it is believed that the viral RNA can evade these internal vesicles and reach the cytoplasm through membrane pores (Welsch and Zeuzem, 2009). With this knowledge, we decided to investigate whether DENV could also exploit the EV pathway to favor the propagation of its life cycle, as has been shown for other viruses.

### EVs Secreted by Infected Cells in a DENV Model Can Carry Viral Components and Transfer Infectivity

Previous studies have demonstrated the association of infective viral particles with EVs. For HCV, it has been shown that CD81 is linked to infective particles (Fénéant et al., 2014), while yellow fever virus requires Alix (a protein enriched in EVs) for its budding, suggesting an overlap with EV release pathways (Carpp et al., 2011). For HIV, it was observed that a subpopulation of viruses produced by infected macrophages is associated with EVs and can infect T CD4 cells more efficiently than stock viruses (Kadiu et al., 2012). Additionally, for pegivirus, it was shown that EVs from patient serum could carry viral RNA and transfer the infection to human PBMCs (Chivero et al., 2014). In addition, HCV RNA associated with Ago2, HSP70 and miR-122 can be transferred through EVs, generating productive infection (reviewed by Chahar et al., 2015). Also, it was already shown that DENV could hijack mast cell extracellular granules, facilitating its spread through the body (Troupin et al., 2016). In addition to the direct transfer of infectivity, EVs can be used to render previously non-permissive cells susceptible to infection, as was shown for HIV through the transfer of the co-receptors CCR5 and CXCR4 (Mack et al., 2000; Rozmyslowicz et al., 2003).

**FIGURE 5:**
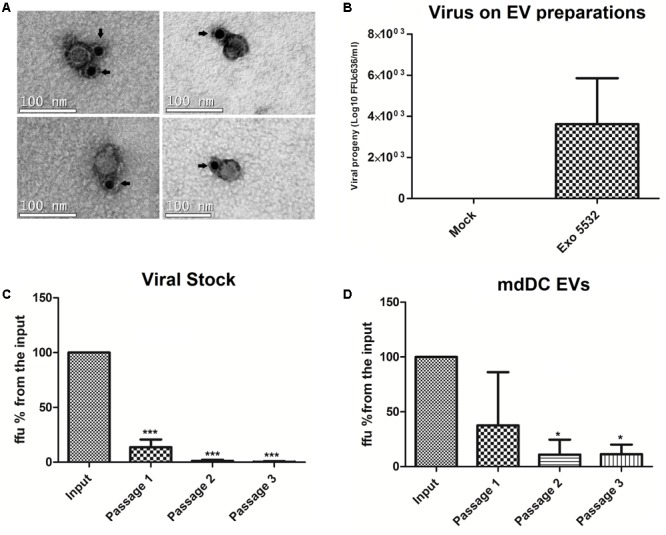
Sequential depletions with 4G2 conjugated microbeads can eliminate the infective potential of viral stocks, but not from infected mdDC derived EVs. Samples that were submitted to one, two or three passages through 4G2 conjugated beads were titrated in duplicates, and the media of duplicates was used for each sample. Three independent depletion experiments were carried. The amount of ffu observed on the input sample was normalized to 100%, and all the samples were compared against the input. **(A)** Particles positive for surface E protein staining, (possible viral particles) are shown to co-precipitate with EV isolates. The antibody staining with gold particles is indicated by arrows. **(B)** Viral progeny showing the amount of viable virus (Log10 FFU c636/ml) detected in different isolations of EVs from infected mdDCs before depletion. **(C)** Depletion assays with stock DENV3-5532. Three different samples were submitted to 2 or 3 passages in the beads, and the viral progeny found for these samples was statistically significant when compared with input sample (^∗∗∗^*P* < 0.0001). **(D)** Depletion assays with EV samples isolated from mdDCs infected with DENV3-5532. Each sample was formed with pooled EVs from infected mdDCs from three different donors, totalizing nine donors. Samples submitted to 2 or 3 passages in the beads were statistically significant when compared with input sample (^∗^*P* < 0.05). Vesicles submitted to two or three rounds of depletion do not have statistical differences between its infective potential, but still keep its infectivity, suggesting that a subset of viable viruses is in someway protected from antibody binding.

To investigate whether DENV particles could be associated with EVs and exploit the EV pathway for its own survival, we first performed a structural analysis of EV isolates by electron microscopy. EVs were isolated from infected primary human mdDCs and stained with an antibody that recognizes a DENV envelope protein conjugated with gold particles. We could observe particles that were smaller than the average EV (approximately 50–60 nm) with a consistent round morphology and which were positive for the viral E protein staining (**Figure 5A**), suggesting the co-precipitation of viral particles with EVs in these samples. To confirm whether these particles had infective potential, EVs derived from infected mdDCs were titrated (focus forming assay, FFU_C6/36_/mL) in C6/36 cells, as previously described. The results demonstrated that EVs from DENV3-infected mdDCs carry approximately 10ł–10^4^ FFU_C6/36_/mL of DENV (**Figure 5B**), showing that the EV samples had potentially infective viruses.

Subsequently, a protocol to separate the EV populations from the viral particle populations was developed. A previously published iodixanol-based separation protocol with several modifications was used (Pegtel et al., 2010); however, it was not successful in separating infective DENV particles from EVs (data not shown). To further investigate whether these viable viral particles could be removed from the EV pool, a depletion protocol using 4G2-conjugated beads was developed in order to capture infective viral particles and eliminate them from the EV preparation. The assay was first standardized with viral stocks of DENV3-5532. Different concentrations of beads and 4G2-antibody were tested (1 × 10^9^ beads/20 μg 4G2-Ab, 2 × 10^9^/40 μg, 4 × 10^9^/80 μg) with no increase in depletion efficiency for each passage in the beads (data not shown). Thus, we chose to use the smaller amount of beads/antibody (1 × 10^9^/20 μg of antibody) and to pass the samples successively through the beads in an attempt to progressively remove the infective DENV particles from the EV samples. Magnetic beads conjugated with 4G2-antibody were incubated with aliquots of the viral stocks containing 10^4^ FFU_C6/36_ per sample, since all the samples of mdDC derived EVs had less viral particles than 10^4^ FFU_C6/36_ (**Figure 5B**). After the incubation, the supernatant was separated and further incubated with fresh 4G2-conjugated beads for three rounds of depletion. For each passage, an aliquot of the supernatant was collected and the samples were titrated. We observed that for viral stocks, the samples submitted to two passages in the beads showed an infective potential of less than 1% of the input virus, and three passages in the beads were enough to eliminate the infective potential of the samples (**Figure 5C** and Supplementary Figure S5). This preliminary result indicated that this depletion protocol was able to eliminate the viable DENV viral particles from the DENV stocks.

With the protocol standardized for viral stocks, we isolated new EVs from infected human mdDCs, and the same depletion protocol was applied to the EV preparations. Interestingly, the results observed were different; in the first round of depletion the amount of viral particles in the mdDC-derived EV preparations decreased (**Figure 5D**). However, for the remaining rounds of depletion, the amount of viral particles in the samples remained constant and did not decrease further with additional passages (**Figure 5D**), and no statistical differences were found between samples submitted to two or three rounds of depletion. This result suggests that free viral particles were present in the EV samples and that some viral particles appear to be protected from capture by the antibody due to association with or internalization in the EVs. There is also a possibility of the vesicles carrying infective DENV RNAs without carrying the full viral particles, as already shown for HCV (Ramakrishnaiah et al., 2013), and this could be further explored in the future. In these experiments we show that, even when removing free viral particles by antibody binding, the infective potential of DENV derived EVs is maintained, suggesting that the EV preparations can protect viral particles or infective RNA in some way. From these experiments and from previously data, we hypothesize that a subpopulation of DENV exists as free viral particles secreted on cell supernatants, which coprecipitate with EVs. We showed this first in **Figure 5B**, when it is possible to observe that vesicle preparations were able to infect C636 cells, and this infection would partially decrease if EV preparations were passed in beads conjugated to 4G2 (**Figure 5**), suggesting a subpopulation of free viral particles. Also, we can see free particles resembling DENV, stained with 4G2 on EM. However, we believe that a subpopulation of viral particles (or vesicles carrying viral RNAs) could be somehow protected when associated with EVs, since bead depletion was not completely efficient to eliminate the viruses from mdDC derived EVs. This experiment also shows that the protected virus particles are infective, since the virus was detected by titration (infection of mosquito cells) suggesting that DENV can exploit the EV pathway to propagate its infectivity to other cells, evading immune surveillance.

## Conclusion

The role of EVs during viral infection has been previously described for several viruses, such as HIV, hepatitis viruses, poxvirus and Epstein-Barr virus (reviewed by Meckes and Raab-Traub, 2011). In addition to the great importance of EV pathways for the transfer of immune signals that can help to counteract infection, they can also be exploited by viruses to facilitate their own survival or manipulate host immune responses (Wurdinger et al., 2012). In this way, EVs can promote cellular internalization of viruses, viral evasion and enhancement of viral replication (reviewed by Meckes and Raab-Traub, 2011; Hosseini et al., 2013). Dendritic cells are the first site of DENV replication; therefore, modulation of dendritic cell gene expression in the first moments after infection could generate EVs loaded with molecules that, once transferred to neighboring cells, make them more permissive to infection. On the other hand, antiviral responses triggered by infected cells could produce “protective EVs” that could elicit a protective response when released to neighboring cells, triggering an antiviral response even before the viruses reach them. It is known for other viral models that both types of EVs can coexist *in vivo*, and the dynamic balance between “protective EVs” and “virus-manipulated EVs” help dictate the infection outcome (Alenquer and Amorim, 2015).

In addition to transferring host RNAs and proteins between cells, EVs can also carry viral components. The HTLV1 proteins Tax, HBZ and Env have been shown to be associated with EVs (Sampey et al., 2014; Chahar et al., 2015). Transcripts coding for viral proteins were also detected in EVs secreted by infected cells, as was shown for HTLV1 Tax mRNA (Jaworski et al., 2014), HCV Core, APOe and APOb mRNAs (Ramakrishnaiah et al., 2013) and for GAG and NEF mRNAs from HIV (Aqil et al., 2014). The viral micro-RNAs vmiR88, vmiR99, and vmiR-TAR were also detected in EVs produced by HIV-infected cells (Bernard et al., 2014). In addition, it has been shown that human epithelial cells can secrete antiviral components that inhibit the infection of macrophages with HIV (Guo et al., 2018).

Here, we have shown that infection by DENV can modulate the content of EVs secreted by primary dendritic cells and that DENV possibly exploits the EV pathway for its own benefit. Cells infected with two different strains of the same serotype produced vesicles with dramatically different RNA contents. A strain isolated from a severe fatal case can induce the production of EVs packed with RNAs associated with inflammatory responses, which was not observed for another strain of the same serotype isolated from a non-severe case. These results agree with previous works showing that dengue viruses from the same genotype with small differences at the genome level can induce different cellular responses, reinforcing the possible role of viral components in inducing a strong immune dysfunction during severe dengue infection.

Additionally, some of the mRNAs found in the EVs were previously demonstrated to be expressed in mdDCs infected with the same DENV3 strain. Thus, the EV pathway can transfer different immune signals during distinct infectious conditions. We also suggest that EV RNAs should be further explored as a potential biomarker of dengue severity, since their circulation in the bloodstream and other biofluids would allow a quick detection, which is important for the development of severity prognostics in dengue.

We also showed that vesicles from infected cells can carry mRNAs of interferon stimulated genes and further confirmed that vesicles from IFN-treated cells can have a protective effect against the infection, successfully blocking the infection in some of the neighboring cells, which suggests that signals involved in the IFN-mediated response can be carried through EVs and be protective during DENV infection, as immune cells communicate through the EV pathway.

In addition to the effect on immune cell communication during DENV infection (that could enhance and propagate the host antiviral response), we also obtained evidence that in some way, association of DENV with EVs can protect its infectivity potential. We showed the association of infective DENV particles with EVs, and this association could not be completely removed by antibody binding, suggesting that EVs could protect infective viral particles in such a way that they can hide from immune surveillance, favoring viral propagation.

The potential of immune cell-derived EVs and the miRNAs that they carry should be further explored for the development of prognostic tools that can predict the development of severe dengue cases.

## Ethics Statement

This study was carried out in accordance with the recommendations of Fiocruz Research Ethics Committee. The protocol was approved under the number 514/09. All subjects gave written informed consent in accordance with the Declaration of Helsinki.

## Author Contributions

STM did most of the experiments, discussed the results, and wrote the manuscript. DK collaborated in the experiments and discussion of the results and in the critical reading of the manuscript. LA collaborated in the RNA sequencing experiments and analysis, discussion of the results, and critical reading of the manuscript. JL and JB collaborated on the discussion of the results, and critical reading of the manuscript. All authors approved the submitted version.

## Conflict of Interest Statement

The authors declare that the research was conducted in the absence of any commercial or financial relationships that could be construed as a potential conflict of interest. The reviewer JM and handling Editor declared their shared affiliation.
