# Supplementary material for: Characterization of Dendritic Cell-Derived Extracellular Vesicles During Dengue Virus Infection
Source: Front Microbiol. 2018 Aug 6;9:1792. doi: 10.3389/fmicb.2018.01792 (PMC6090163; doi:10.3389/fmicb.2018.01792)
Supplement: Supplementary file 6 [file Data_Sheet_1.docx]

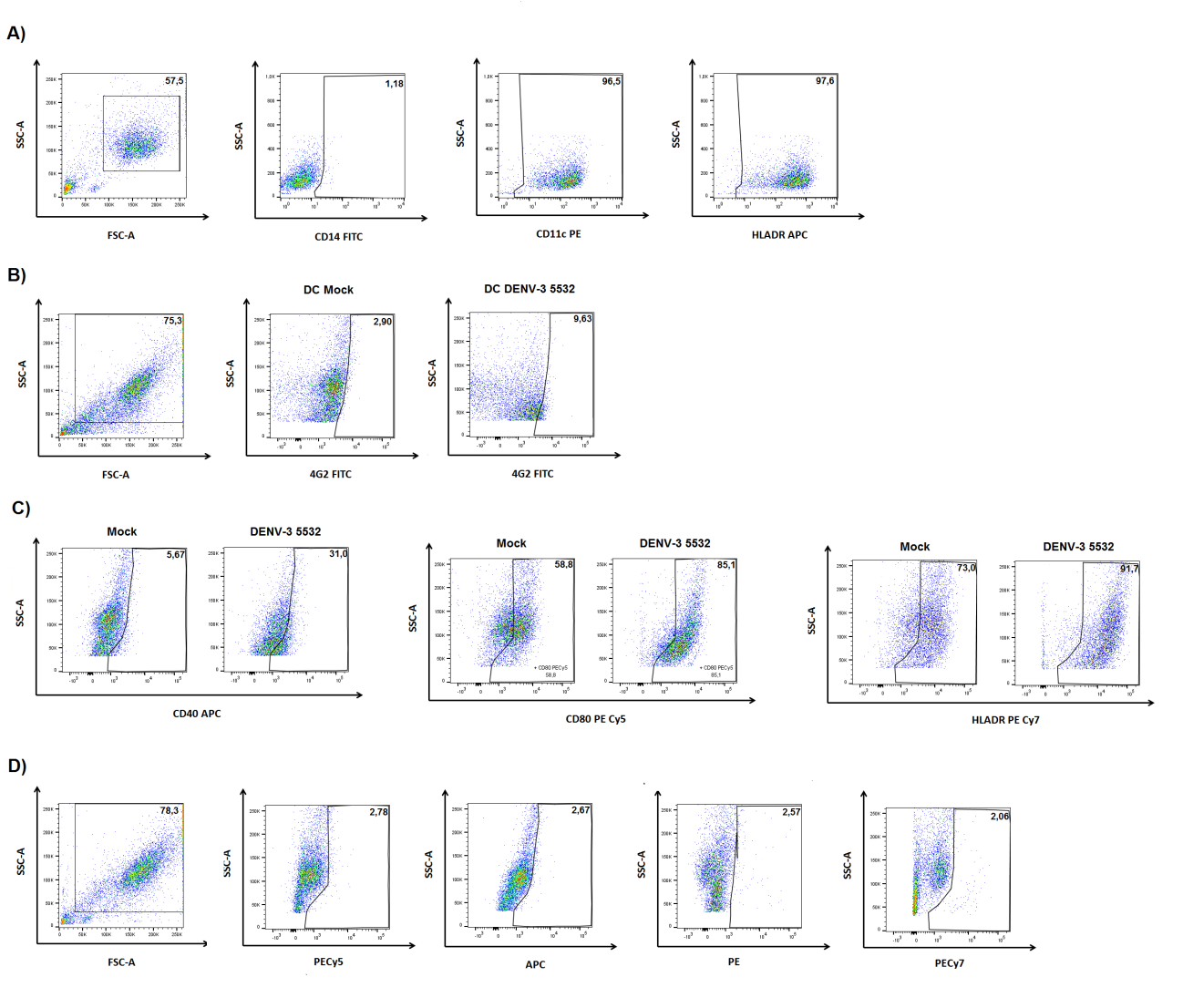


**Supplementary figure 1 -** **A)** **Representative dot plots showing the expression of CD14, CD11c and HLADR on monocyte-derived dendritic cells**. The gates were delimitated using isotype controls for each antibody, as shown in S1 C. **B)** **Representative Plots showing intracellular staining of viral E protein in mdDCs**. Left panel: gate delimiting the mdDCs population; center panel: E protein staining of Mock cells; Right panel: E protein staining of mdDCs infected with DENV3-5532**. C)** **Representative Plots showing the expression of CD40, CD80 and HLADR for cells infected with DENV3-5532 (MOI10, 72h) and Mock. D)** **Isotype controls for the fluorophores used.**


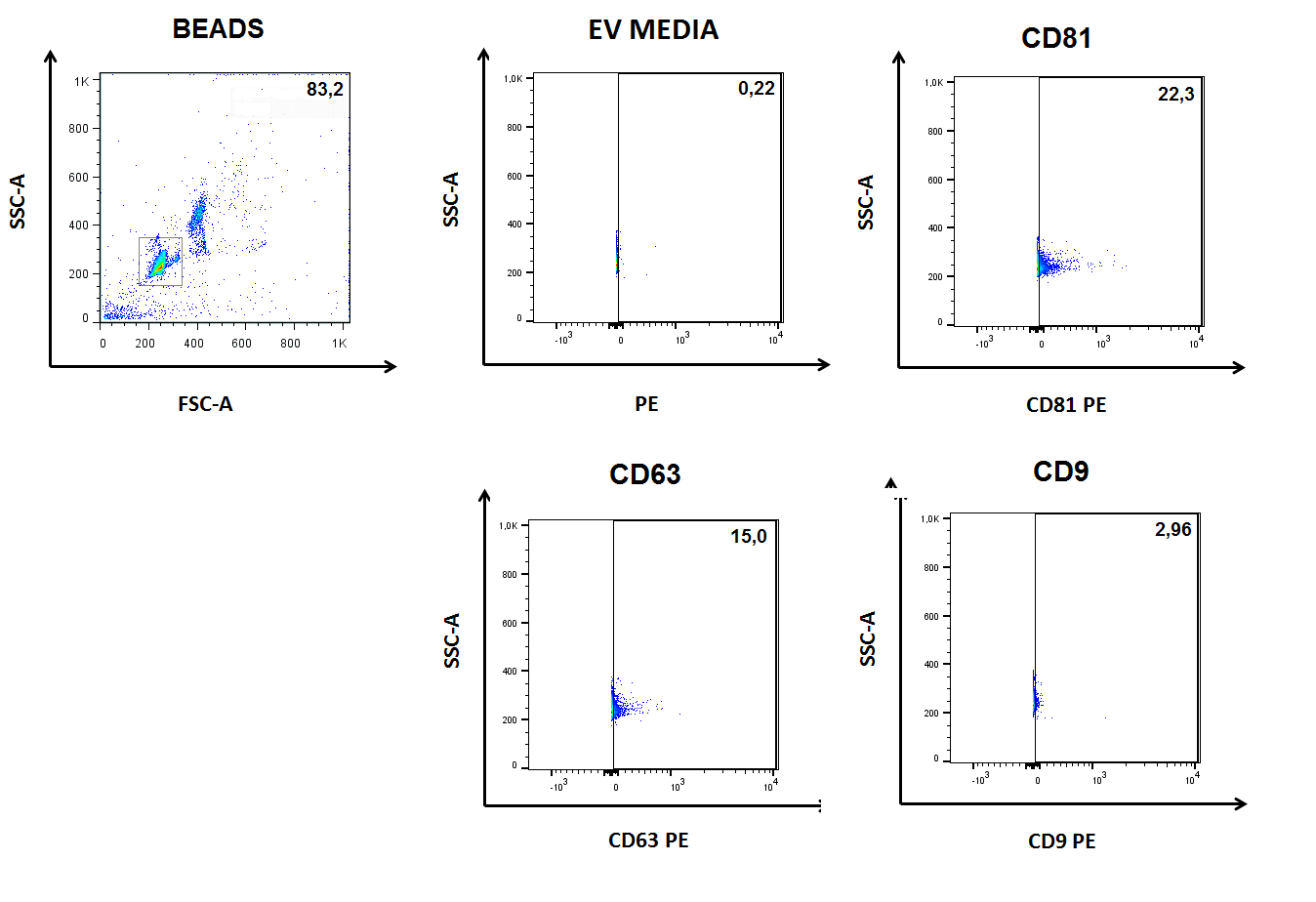


**Supplementary figure 2 - Representative plots showing the expression of CD9, CD63 and CD81 in EVs captured with latex beads**. Gates were delimitated with microspheres bound to samples and stained with isotype antibodies conjugated with PE.


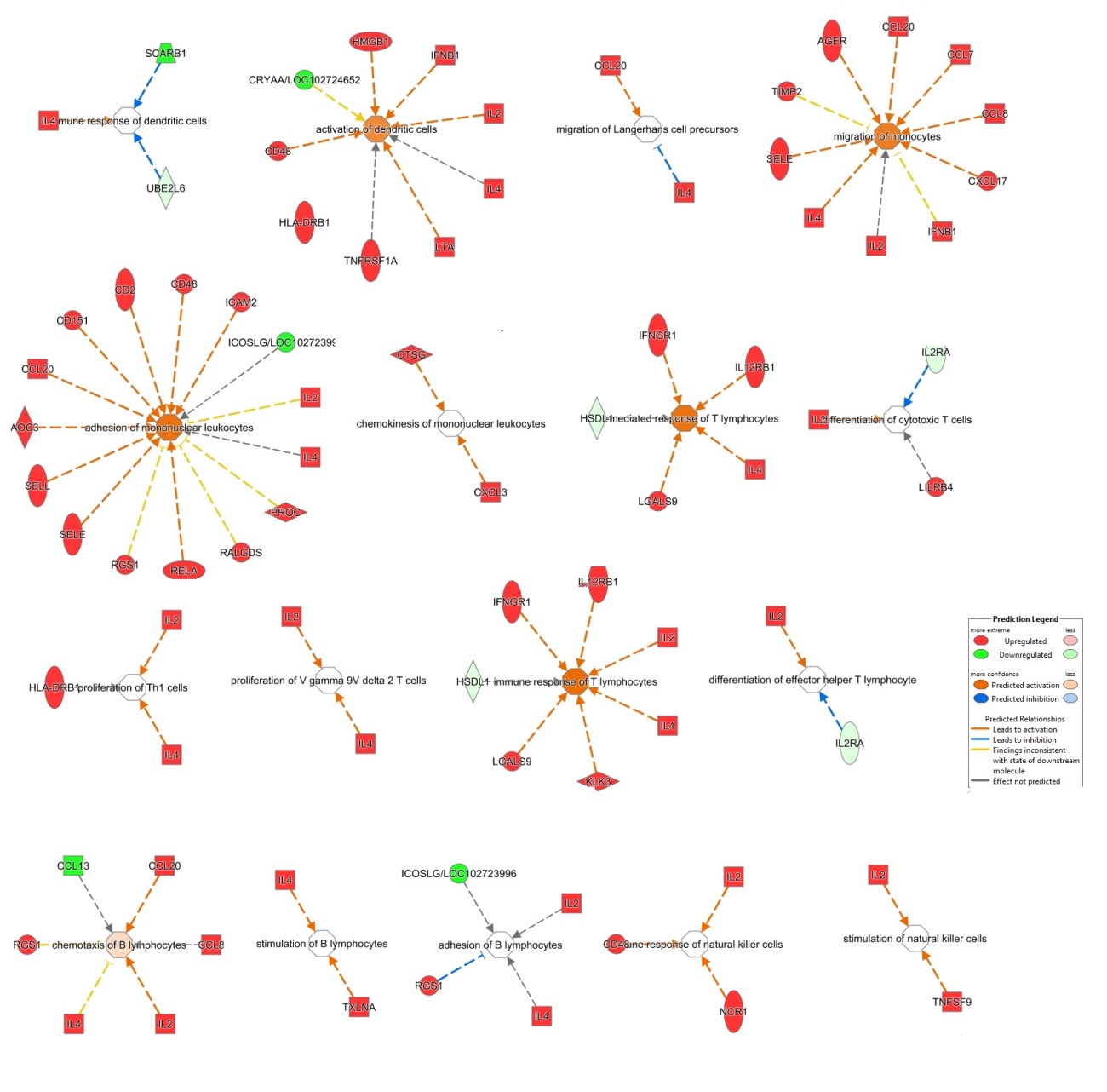


**Supplementary Figure 3 – IPA Pathways related to immune responses for mRNAs found in DENV3-5532 and DENV3-290 EVs**. mRNAs marked in red are more expressed in DENV3-5532 EVs, while those marked in green are more expressed in DENV3-290 EVs. Arrows indicate the putative action of those genes over the pathways – orange arrows indicate an activator role, while blue arrows indicate a repressive role


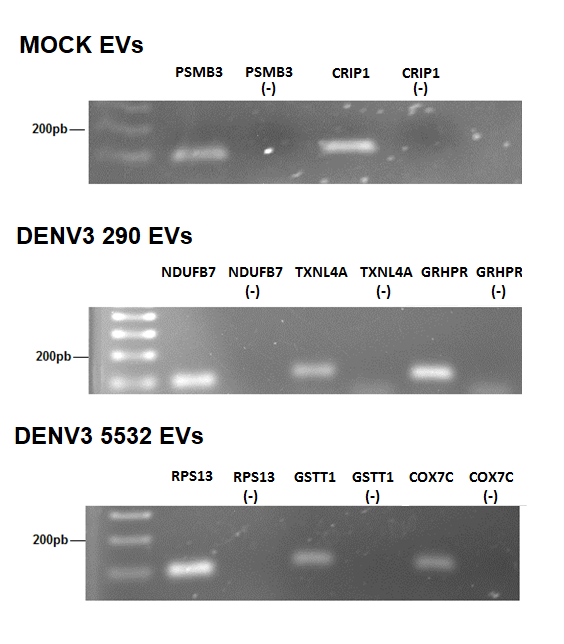


**Supplementary Figure 4 – Eletrophoretic profile of amplicons obtained from mdDC EV RNAs, after RT-PCR**. Samples marked as (-) represent RT-PCR negative controls for each specific primer. Marker: 1kb Plus (invitrogen)


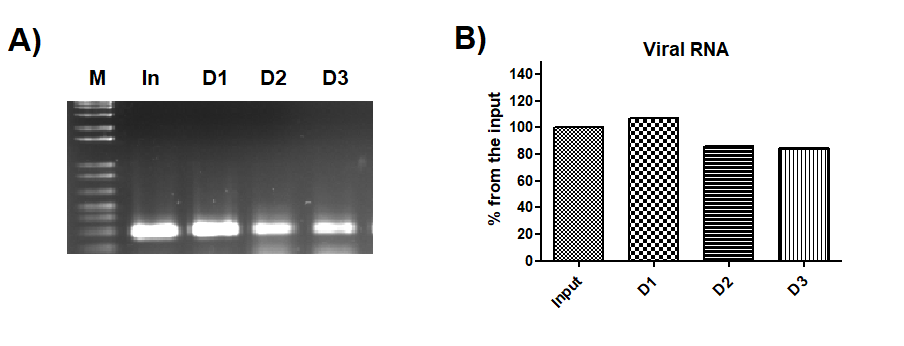


**Supplementary figure 5 - Evaluation of depletion efficiency of DENV from mdDC-EV pools by RT-PCR.** Agarose gels stained with ethidium bromide showing amplification of a DENV3-specific fragment. In: input EVs before depletion. B1, B2, B3: supernatants or first, second and third rounds of depletion, respectively

**Supplementary table 1 –** miRNAs found in EVs secreted by mdDCs infected with DENV3- 5532, DENV3-290 or by mock cells, collected 72h post infection

**Supplementary table 2 -** Putative targets of EV miRNAs in recipient cells predicted in DIANA mirPATH

**Supplementary table 3 -** Subset of mRNAs used as input for Ingenuity Pathway Analysis

**Supplementary table 4- Ingenuity Pathway Analysis of EV mRNAs in the Diseases and Functions mode**

**Supplementary table 5 - Ingenuity Pathway Analysis of EV mRNAs in the Canonical Pathways mode**
